# Investigation of the Experiences of Mothers Living Through Prenatal Loss Incidents: A Qualitative Study

**DOI:** 10.1097/jnr.0000000000000289

**Published:** 2019-05-20

**Authors:** Ruveyde AYDIN, Öznur KÖRÜKCÜ, Kamile KABUKCUOĞLU

**Affiliations:** 1 RN, Doctoral Candidate and Research Assistant, Faculty of Nursing, Department of Gynecology and Obstetrics Nursing, Akdeniz University, Antalya, Turkey;; 2 PhD, RN, Assistant Professor, Faculty of Nursing, Department of Gynecology and Obstetrics Nursing, Akdeniz University, Antalya, Turkey;; 3 PhD, RN, Professor, Faculty of Nursing, Department of Gynecology and Obstetrics Nursing, Akdeniz University, Antalya, Turkey.

**Keywords:** termination, pregnancy loss, motherhood, abortion, Turkey

## Abstract

**Background:**

Fetal death causes women to experience negative feelings after the loss. These lived experiences influence the future pregnancies and maternal health of women negatively.

**Purpose:**

The aim of this study was to investigate the experiences of women whose pregnancies were terminated because of medical indications.

**Methods:**

A “design for definitive status study” type of qualitative research design was used. Ten women who were hospitalized between April and July 2017 at the Akdeniz University Clinic of Obstetrics and Gynecology and had subsequently experienced pregnancy loss during their hospitalization were enrolled as participants. Thematic analysis was used to analyze the data.

**Results:**

The pregnancy loss experiences of participants were investigated under the five main themes of (a) lived experiences before the termination of pregnancy, (b) lived experiences after pregnancy termination, (c) willingness to see the baby after termination, (d) posttermination care requirements, and (e) physical condition of hospital rooms during hospitalization. The participants expressed feelings of hope, fear, and worry over being unsuccessful before fetal death and feelings of loneliness, disappointment, blame, and avoidance after fetal death.

**Conclusions/Implications for Practice:**

In the aftermath of fetal death, health professionals should use insightful and empathic communication skills to help mothers cope with their loss. In addition, some of the participants wanted to see their babies, and some did not. Thus, individualized care is very important for women who experience pregnancy loss.

## Introduction

Despite major advancements in medicine and obstetrics, women are still frequently faced with undesired conditions such as aborted fetuses, dead births, and neonatal deaths, which deeply affect the expectant mother and her family (Meredith, Wilson, Branjerdporn, Strong, & Desha, 2017). Nearly half (45%) of deaths below the age of 5 worldwide are neonatal deaths in 2016. In addition, 2.6 million babies die in the last 3 months of pregnancy or during childbirth (stillbirths). However, 75% of newborn deaths may be prevented through high-quality care (World Health Organization, 2016). According to the Turkey Demographic and Health Survey in 2013, the ratio of missed abortion incidents is 23%, the ratio of stillbirths is 3%, and the neonatal death rate is 0.7% in Turkey (T. C. Ministry of Development & T. C. Ministry of Health, Ankara, Turkey, 2014).

Losses experienced due to reasons such as undesired abortion, stillbirths, and neonatal births cause emotional responses such as mourning and depression (Sriarporn et al., 2017; Yilmaz & Beji, 2013). Mourning is always felt for the lost baby, and he or she is never forgotten. After the loss, women experience a sudden emptiness and they mourn (Smart & Smith, 2013). When the baby dies or when the expectant mother is told that her baby will die, she feels herself unsuccessful and guilty (Kutteh, 2014). For the parents making exciting and hopeful plans for the baby’s future, a death incident occurring during pregnancy makes them lose their hope and dreams. The period of mourning experienced may increase the perceived trauma of this event (Robinson, 2014). Furthermore, women experiencing the loss think that the underlying reason for termination is their own inability to carry the baby to term due to their acting in a manner that harmed the fetus or to their neglectful behavior during prenatal care (Jones, Baird, & Fenwick, 2017). Because parents believe that the death was avoidable, they think that they are responsible for the baby’s death (Jones et al., 2017). This situation causes feelings of guilt and failure to fully recover from the trauma (Jones et al., 2017; Kristjansdottir & Gottfredsdottir, 2014).

Emotional responses to the loss vary depending on the strategies used to deal with it (Jones et al., 2017) as well as on family structure, economic status, past psychiatric history, insufficient social support, not having any living children, insufficient information about pregnancy and loss, and having cultural, spiritual, and religious beliefs (Jones et al., 2017; Meaney, Corcoran, Spillane, & O’Donoghue, 2017). The literature shows that psychological problems experienced by women who experience pregnancy loss include fear, disappointment, anger, self-pity, insufficiency, failure, social isolation, despair (Jones et al., 2017; Kristjansdottir & Gottfredsdottir, 2014), guilt, hopelessness, and depression (Kristjansdottir & Gottfredsdottir, 2014; Meredith et al., 2017).

Understanding the experiences of women who experience pregnancy loss may help healthcare professionals prepare appropriate psychosocial support, support groups after the loss incident, and care guides that enhance quality of care. In addition, this support may help healthcare professionals meet the expectations of immediate family members and support the women to cope with the long- and short-term effects of the loss. The aim of this study was to clarify the experiences of women who have lived through pregnancy loss due to terminations resulting from medical indications.

## Methods

### Research Design

As similar studies have rarely been conducted in Turkey, this study adopted a thematic analysis approach and a qualitative and definitive qualitative research design. Definitive status research is a method that is recommended for researchers who wish to investigate case-specific factors and to comprehensively assess the impact of these factors on participants (Creswell & Poth, 2017).

### Participants

The sample group comprised women who were hospitalized between April and July 2017 at the Akdeniz University Clinics of Obstetrics and Gynecology after pregnancy loss and who volunteered to participate. The women who were included as participants were chosen using the criterion technique, a form of purposeful sampling (Marshall & Rossman, 2015). The sampling criteria in the study were as follows: (a) pregnant women over the age of 18 years and below the age of 45 years, (b) free of chronic and psychiatric diseases, (c) hospitalized because of pregnancy termination and without medical complications during hospitalization, (d) able to communicate in Turkish, and (e) willing to participate in this study voluntarily.

In qualitative studies, saturation point is used to determine the minimum sample size (Morse, 2015). The interview phase of this study was terminated when information provided in the current interview substantively repeated information already obtained in previous interviews. In this study, interviews were conducted with 10 participants.

### Data Collection

Data for the study were gathered using the personal information form and semistructured form that were prepared by researchers. The personal information form comprised 10 questions addressing the sociodemographic features and obstetric information of the participants. The semistructured interview form, which was prepared by the researchers based on the literature, comprised 12 questions (Table 1). Before starting to interview the participants, the opinions of two experts were solicited for the semistructured interview form.

**TABLE 1.**
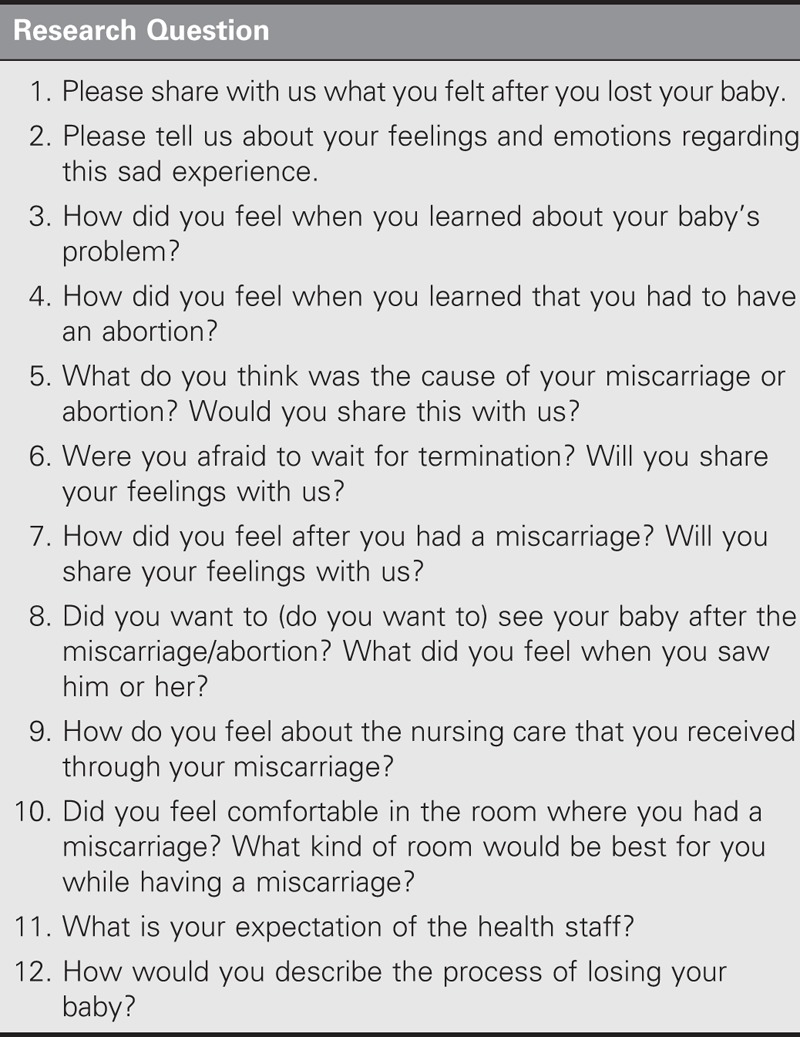
Semistructured Interview Form

Before the interview, the interview room was prepared by the researchers and a notice about the interview was put on the door. The one-on-one, in-depth interviews were conducted in the participant’s room if she was staying in a single room in the hospital or in an empty room in the same ward if she was staying in a multiple-occupant room. The interviews were conducted by one researcher. After all of the questions on the personal information form had been asked and answered, the participant was then asked, “Can you share with us what you felt after you lost your baby?” Subquestions were concurrently asked to enable the participants to relate their experiences and to provide encouragement. An example of these subquestions is, “Can you tell us about your feelings and emotions regarding this sad experience?” Data were collected using a voice recorder. The interviews were conducted at a time that suited the participants and lasted 20–25 minutes for each participant.

### Data Analysis

Thematic analysis is a qualitative analysis method that is used to enable a data set to be organized and explained in detail (Vaismoradi, Turunen, & Bondas, 2013). In this study, the thematic analysis approach of Braun and Clarke (2014) was used, comprising the following six steps: (a) becoming familiar with data, (b) generating initial codes, (c) searching for themes, (d) reviewing themes, (e) defining and naming themes, and (f) producing the report.

### Validity and Reliability

The rigor and trustworthiness of qualitative research are provided through credibility, transferability, dependability, and confirmability (Marshall & Rossman, 2015). In this study, integrity was provided by controlling the relationship among the themes and subthemes that were obtained for credibility. After the interviews with the participants were completed, tape recordings were transcribed verbatim, with the facial/bodily expressions of participants described by two researchers independently and without additional interpretation as to the intent or meaning. Furthermore, the researchers used member checking within the study to enhance the credibility of the transcript. For transferability (applicability), a sampling method with purpose was used and homogeneity was considered. For dependability (consistency), analysis of data was independently conducted by two researchers and the reliability of findings was controlled. During the coding process, opinions were exchanged by the researchers regarding the compliance of the codes that were obtained from data and repeated multiple times. For confirmability (verifiability), the comprehensive interview form and final version of thematization were evaluated by an expert.

### Ethical Considerations

Approval was obtained for this study from the Akdeniz University Clinic Research Ethical Council (Approval no. 742). After potential participants were informed about the study purpose, about how it would be conducted, and that interviews would be recorded using tape recorders, those still willing to participate provided their written and oral consent.

## Results

### Demographic Information

The ages of participants ranged between 25 and 42 years, and most had either a high school or undergraduate education. Four of the participants experienced pregnancy loss in the third trimester, three experienced their loss in the first trimester, and three experienced their loss in the second trimester. From the participants’ hospital records, the reasons for termination included preeclampsia, cervical insufficiency, chromosomal abnormality, and fetal arrest. The pregnancies for nine of the 10 participants were desired and planned. Most participants (*n* = 6) had never given birth, with the remaining four having one living child each.

### Pregnancy Loss Experience

The experiences of the participants during pregnancy termination were investigated under five main themes: (a) lived experiences before the termination of pregnancy, (b) lived experiences after pregnancy termination, (c) willingness to see the baby after termination, (d) posttermination care requirements, and (e) physical condition of hospital rooms during hospitalization (Table 2).

**TABLE 2.**
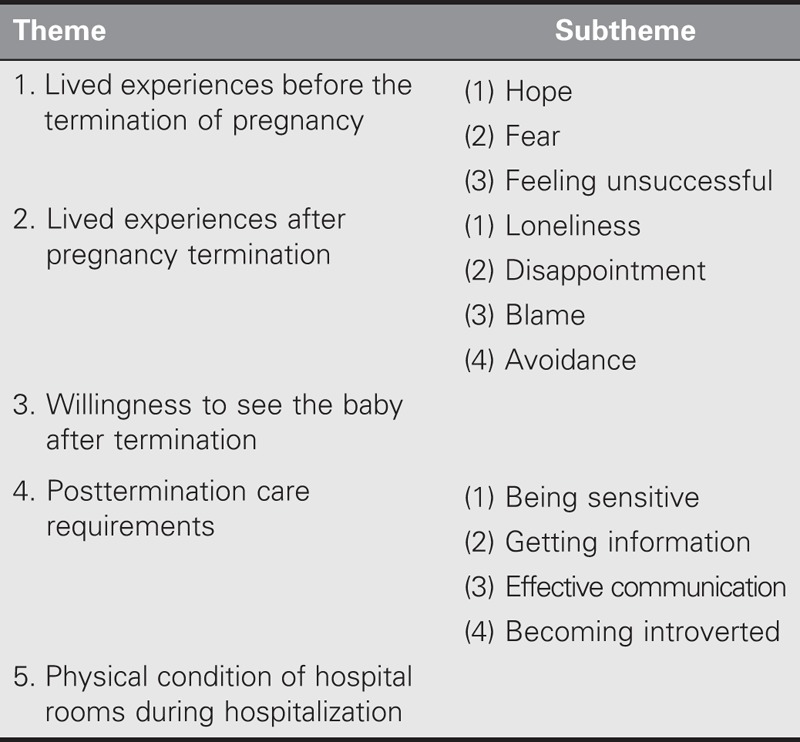
Themes and Subthemes of Pregnancy Loss Experience

#### ***Lived experiences before the termination of pregnancy***

This theme was investigated through three subthemes.

##### Hope

Some of the primiparous participants expressed that they had hoped that their baby might survive even after learning about the problems:

*I used to go regularly for examinations. In the 30th week, they said that his bowels were outside, but that there was a possibility for them to get inside and that they would be following it up. In the 37th week, they said that his heart had stopped and that birth should take place. What could we feel? We were feeling fear and suspicion since the 6th month, but we also had hope.* (M7)

One of the participants who had experienced her third pregnancy loss due to cervical insufficiency expressed what she felt when she learned about the problem with her baby:

*I knew about it since I was three-months pregnant. Suturing was made to by the bottom part (cervix). You are trying to have hope. The doctor said: “We will also suture from the inside. He will have a chance to survive.” And we waited with hope.* (M8)

##### Fear

The fear experienced by participants was related to situations such as repeated incidences of pregnancy loss, being a mother at an advanced age, and not having any living babies. Two of the women, whose first pregnancies had ended up with loss, expressed their experiences as they learned about the problems with their babies:

*I cried a lot. I felt too sad. I wanted to have this baby so much. My age is already advanced. I feared to lose this baby, thinking that I may not have a baby again….* (M9)

*In fact, due to my bleeding and continuous pain, I always had the fear of losing my baby. The baby was always moving and I adapted to it so much, and I felt very afraid to lose him due to this reason....* (M5)

##### Feeling unsuccessful

Some of the participants expressed that they perceived themselves as unsuccessful when they learned about the problem with their baby or when they learned that the baby needed to be aborted.

A participant who had experienced multiple pregnancy losses related her experience upon hearing that her baby needed to be aborted:

*I said that it wouldn’t happen again and that we couldn’t succeed again. Everyone was at home and, as you come from hospital, they are awaiting to hear news from you. It is very difficult to say that it would happen again. You feel yourself unsuccessful in the eyes of your spouse and your family.* (M6)

A primiparous participant who thought that she would live through her pregnancy with treatment related her experience when she learned that her baby needed to be aborted:

…*What can a person feel about it? You are getting good news and then, before reaching to the outcome, you are unsuccessful*.... (M10)

#### ***Lived experiences after pregnancy termination***

This theme was investigated through four subthemes.

##### Loneliness

Several of the primiparous participants expressed that they felt lonely after pregnancy loss, equating their experience to losing a personal possession:

*I felt bad about it. The living creature inside of me was gone, and I was left so alone….* (M1)

*I felt so alone, as if someone I loved so much has left me and gone to a faraway place from where he would not come back again....* (M9)

##### Disappointment

Several of the participants related their disappointment after pregnancy loss as a failure to fulfill their dreams:

*We had prepared our baby’s room. I had dreamed about buying clothes and doing shopping for him, but it couldn’t happen now....* (M8)

*When I learned about the problem, I consoled myself that the pregnancy would continue and I felt that this could happen. But it turned out to be disappointing for me. I had such dreams with my spouse....* (M10)

##### Blame

Two of the participants believed that their pregnancy loss occurred because of their own circumstances and that they felt guilty about it:

*This loss occurred due to an infection. I blame myself so much. Thinking that I had no complaints, I did not use my infection drugs. I am the only one responsible for this loss. I will never forgive myself.* (M5)

*It is as if something you owned has left you and that you are the person to be blamed most for this leaving….* (M6)

##### Avoidance

A participant who experienced pregnancy loss due to cervical insufficiency expressed how she could overcome the loss through socializing:

*The period following the loss is a difficult time. You have to socialize to forget…. You concentrate more on your spouse, your friends, and your work. You change the center of your life.* (M2)

#### Willingness to see the baby after termination

Although most of the participants (*n* = 6) did not want to remember their babies or to see them in their dreams, several stated that they wished to see their babies after the pregnancy loss.

One participant who did not wish to see her baby after the loss incident said:

*I didn’t want to see my baby. If I had seen him, I wouldn’t forget him. Even though I didn’t see him, I still see him in my dreams. He is coming out of my stomach and flying away. I try to catch him, but can’t. It is so painful…*. (M1)

A minority of the participants wished to see their babies. One woman who had experienced multiple (four) pregnancy losses expressed that she wished to see her baby:

*Obstetricians did not want to show him to me but I insisted. I thought I had all this pain for nothing in the end. It was my right to see him. My baby was lying with his head turned to the side just like an angel.* (M2)

One of the participants who self-described as being of advanced age told about her reason for wishing to see her baby:

*I wanted to see my baby. As I am advanced in age, I thought that, if I don’t have a baby again, I would still live with his dream. He was so beautiful. My baby was just like an angel…. I never wanted to leave him and it was the most difficult moment on earth….* (M9)

#### ***Posttermination care requirements***

The opinions of the participants regarding this theme were investigated through four subthemes.

##### Being sensitive

Many of the participants related that, during the loss period, doctors should make a concerted effort to understand them, that the period being lived through should not be normalized, and that motherhood is a special life experience:

*It is as if doctors are too insensitive. They act as if what is being lived through is something very normal. They say that we can try again. They talk as if the period being lived through is very simple. They should be more sensitive.* (M1)

##### Getting information

A number of participants stated that they faced problems during the loss period in obtaining sufficient information and that being informed during this period is very important.

*Doctors make people wait for a long time during this period. You want to get information about your baby but you cannot. They should be more sensitive and they should give information. I would die of heart attack when I was waiting.* (M7)

One of the participants who had experienced multiple pregnancy losses stated that she had problems reaching her doctor when she wanted:

*I should be able to reach my doctor when I want, and I should be able to get information when I want. I call him but he does not answer. He does not answer my messages. You are living through a difficult period and you are telling yourself that there is loss and that it shouldn’t have happened again….* (M2)

##### Effective communication

Several of the participants related that, although they were content with the way nurses communicated, they were not content with the way their doctors communicated:

*The nurses couldn’t be any better. They are very involved, interested and pleasant.... But the doctors don’t even look in the face of the person when they are talking. I cannot communicate with my doctor much and I cannot even see him....* (M3)

Another participant said:

*Doctors should especially be very pleasant. They should know that there is a person standing next to them and that everyone is precious and they should react to their patients accordingly. But there is a big problem with how doctors communicate. They don’t even look us in the face.* (M5)

##### Becoming introverted

Two of the participants related that it did not matter how health professionals treated them after the loss, as they did not wish to speak and wanted rather to be introverted and to deal with the situation on their own.

*I prefer being introverted. I don’t want anyone to be involved with me. I want to fight with the emptiness in me on my own and, afterwards, I want to get back to my normal life.... For this reason, the care that is given is not important for me….* (M6)

*I want to be introverted and to live through my pain on my own. My only wish is for everyone to keep away from me....* (M10)

#### ***Physical condition of hospital rooms during hospitalization***

All of the participants had requested accommodations in single rooms after their loss and expressed the desire to stay in the room either alone or with their spouses.

*There should be single-person rooms. Because sometimes a person wishes to cry out loud. I am lying on the door side and when people are coming in and out, they are seeing me and I don’t want this to happen when I am crying….* (M7)

Another participant who shared a room with women who had just given birth said:

*I was lying in the middle bed in a room for three people. On the side beds, there were mothers who had newly given birth and who already have children. I felt psychologically bad when I saw them....* (M8)

## Discussion

Women who experience pregnancy loss may experience complex, emotional feelings (Jones et al., 2017; Kersting et al., 2009). The purpose of this study was to clarify the experiences of women who have lived through prenatal loss resulting from a termination prescribed by medical indications. The lived experiences of these women were investigated in accordance with the five main themes of lived experiences before the termination of pregnancy, lived experiences after pregnancy termination, willingness to see the baby after termination, posttermination care requirements, and physical condition of hospital rooms during hospitalization.

The death of a baby is one of the most painful life experiences of patients and is a difficult problem to handle (Meaney et al., 2017). Whether a mother has her baby aborted or whether she is faced with a neonatal death, death means losing the future and the hopes that were associated with the baby (Richards, Graham, Embleton, Campbell, & Rankin, 2015). Especially as the plans of families are suddenly lost, the disappointment and pain of parents can be traumatic (Robinson, 2014). In addition to losing the child, the related dreams of parents, role expectations, visions for family life, and expected milestones are lost as well (Jones et al., 2017; Lafarge, Mitchell, & Fox, 2013). During this period, parents live through a sudden emptiness and feeling of loneliness (Fisher & Lafarge, 2015). In prior studies, mothers who had experienced this loss reported experiencing heavy isolation (Barr & Cacciatore, 2008; Ryninks, Roberts-Collins, McKenzie-McHarg, & Horsch, 2014), despair, loneliness, and disappointment (Meredith et al., 2017; Ryninks et al., 2014). Besides, during the loss period, women may blame themselves and perceive their bodies as unsuccessful, which may lead to discordant behaviors (Cacciatore, 2010b; Lafarge et al., 2013).

After a pregnancy loss, some women want to see their baby and some do not (Andersson, Christensson, & Gemzell-Danielsson, 2014). In this study, the participants who did not want to see their stillborn babies stated reasons including the fear of not forgetting or of remembering their baby, whereas those who wanted to see their stillborn babies stated a desire to face the reality and to say farewell to their pregnancy and baby, which supports the findings of other studies (Stålhandske, Makenzius, Tydén, & Larsson, 2012).

Patient-focused care is critical in the treatment of patients in the period immediately after a traumatic loss such as a stillbirth or neonatal birth (Cacciatore, 2010a). However, health professionals may sometimes medicalize the prenatal loss period from a medical perspective. This situation may cause women to perceive their loss as an emotional symbolic case in which they perceive themselves as being unsuccessful and health professionals as being inconsiderate (Lee & Rowlands, 2015; Simwaka, de Kok, & Chilemba, 2014). This study confirmed that some health professionals normalized the loss incident and that greater sensitivity and communication are needed from health providers in these situations. Prior studies have shown that, in providing care for women experiencing loss, when nurses expressed understanding their patients’ lived experience via empathy for the meanings of pregnancy and motherhood, it made it easier for the patients to deal with the loss incident (Kimport, Cockrill, & Weitz, 2012; Richards et al., 2015; Simwaka et al., 2014). For this reason, communication between the woman experiencing the loss and the care provider is very important.

In this study, the participants expressed a desire to stay in single-person accommodations after the loss incident and that sharing a room with other pregnant women or women who had already given birth negatively affected their psychological well-being. In Meaney et al. (2017), women who had experienced a pregnancy loss expressed that it was difficult for them to stay in the same room with other pregnant women and that they purposefully suppressed their anger and sadness while in shared accommodations. Therefore, with regard to the physical conditions of hospital rooms, women should be allowed to stay in single accommodations after a pregnancy loss and, if not possible, should be assigned to rooms without other pregnant women or women with babies (Andersson et al., 2014; Stålhandske et al., 2012).

### Implications

The lived experience of pregnancy loss affects women both physically and emotionally. Health professionals who provide postloss care should understand the emotional status of the woman experiencing loss, provide appropriate support, and communicate empathetically. Therefore, training should be provided to health professionals who provide care to women experiencing pregnancy loss to improve their empathetic communication skills and their ability to provide appropriate and effective care services after a pregnancy loss.

Mothers experiencing loss require the support of health professionals as well as of social and religious-based institutions. For this reason, perinatal mourning programs should be developed by health institutions and social institutions to provide support and consulting services to parents after a pregnancy loss.

Women experiencing pregnancy loss should stay in single rooms with their families after their loss incident if the physical conditions of hospital permit. If conditions do not permit, these women should be given priority consideration for low-occupancy rooms without other pregnant women or babies.

### Strengths and Limitations

The strongest aspect of this research is that the information on the experiences of women living through the loss incidents was obtained using qualitative interviews, which allowed eliciting the perspective of the participants in an environment that was free of external influences.

As the participants did not wish to review their loss multiple times, they did not want to see their transcript. Thus, the transcripts were not confirmed for accuracy with the participants before analysis.

### Conclusions

Immediately after a pregnancy loss event and regardless of the reason for the loss, women live through feelings of guilt, disappointment, failure, avoidance, introversion, and fear. Health professionals should be more sensitive to the experience of these women and should provide more comprehensive information about the period of loss. Some participants did not wish to see their babies because of fears of not being able to forget them, and others thought that it was their right to see their babies. After a pregnancy loss, the affected mother should not be required to share a room with women who are awaiting or who have just given birth. Thus, after a pregnancy loss, women should be assigned either to single rooms or to rooms without expectant mothers or women with their newborn children.
